# Photobiomodulation for salivary gland dysfunction: aligning clinical expectations with etiology-dependent treatment responsiveness

**DOI:** 10.1007/s10103-026-04991-5

**Published:** 2026-08-19

**Authors:** Caio Camargo Calarga, Caio Bruno Tolentino de Brito, Cláudia Carrara Cotomácio, Alyne Simões

**Affiliations:** 1 Faculdade de Odontologia, Departamento de Biomateriais e Biologia Oral, Universidade de São Paulo, São Paulo, Brazil; 2 Universidade Paulista, São Paulo, Brazil; 3 Grupo de Apoio ao Adolescente e à Criança com Câncer (GRAACC), São Paulo, Brazil

**Keywords:** Photobiomodulation, Hyposalivation, Xerostomia, Sjögren, Chemotherapy, Radiotherapy

## Abstract

To discuss how distinct pathogenic mechanisms underlying salivary gland dysfunction may influence responsiveness to photobiomodulation (PBM) in patients with hyposalivation and xerostomia. A narrative review was conducted focusing on the biological and clinical factors potentially associated with PBM responsiveness across different etiologies of salivary gland dysfunction, including Sjögren’s disease, chemotherapy-induced dysfunction, radiation-induced salivary gland injury, and polypharmacy-associated hyposalivation. Mechanisms related to inflammation, oxidative stress, fibrosis, neurosecretory impairment, residual glandular viability, and persistence of etiological burden across different etiologies of salivary gland dysfunction were comparatively discussed. Therapeutic success assessments were also explored, emphasizing that symptom improvement and enhancement of oral quality of life may occur even in the absence of complete salivary flow normalization. In addition, the currently available evidence regarding PBM responsiveness in distinct etiological scenarios was critically presented, highlighting its heterogeneous and unbalanced distribution across conditions. Future directions involving biologically informed, individualized, and combination-based therapeutic approaches were also discussed. PBM responsiveness should not be interpreted as universally predictable across all etiologies of hyposalivation. Instead, therapeutic outcomes likely depend on several factors. Biologically informed and individualized approaches may contribute to more realistic therapeutic expectations and more personalized management strategies.

## Introduction

Hyposalivation is a multifactorial condition associated with substantial impairment in oral health and quality of life, frequently leading to difficulties in mastication, swallowing, speech, sleep, taste perception, and oral comfort [1, 2]. Beyond its symptomatic burden, reduced salivary flow rate (SFR) may increase the risk of opportunistic infections, dental caries, mucosal injury, and nutritional compromise, particularly in medically complex and aging populations [3, 4]. Despite sharing a common clinical manifestation, hyposalivation should not be interpreted as a single disease entity, but rather as the final outcome of biologically distinct mechanisms involving inflammatory, toxic, neurogenic, vascular, and fibrotic pathways [1, 5].

In recent years, photobiomodulation (PBM) has emerged as a promising non-invasive therapeutic strategy for the management of salivary gland dysfunction [6]. Its proposed mechanisms include modulation of inflammatory mediators, improvement of mitochondrial activity and microcirculation, reduction of oxidative stress, and stimulation of tissue repair [6–8]. Although favorable clinical outcomes have been reported in different hyposalivation settings, the literature remains heterogeneous regarding treatment protocols, outcome measures, and therapeutic responsiveness, often yielding inconsistent or difficult-to-compare results [6, 9].

Many discussions surrounding PBM for hyposalivation fail to consider the profound biological differences underlying salivary dysfunction across etiologies. This perspective may contribute to unrealistic expectations regarding treatment outcomes, inaccurate interpretation of therapeutic success or failure, and challenges in protocol selection and long-term management. Importantly, the likelihood of response to PBM may depend on factors such as the degree of salivary gland damage, the presence of residual functional glandular tissue, the persistence of the etiological insult, and the predominance of acute/inflammatory versus chronic/irreversible destructive mechanisms [10, 11]. All these factors are intrinsically associated with the underlying etiology, suggesting that PBM responsiveness may be influenced by etiology and may not be universally predictable across different biological contexts.

Recognizing variability in expected responsiveness should not discourage the clinical use of PBM. Instead, it reinforces the importance of individualized treatment planning, careful alignment of therapeutic expectations, longitudinal reassessment, and the consideration of adjunctive or combined therapies when appropriate [12]. In many cases, therapeutic success may not necessarily correspond to complete recovery of SFR, but rather to symptomatic improvement, stabilization of glandular function, enhanced oral comfort, and improved quality of life [2, 11, 12].

Therefore, this article discusses how distinct pathogenic mechanisms underlying salivary gland dysfunction may influence PBM responsiveness across different etiologies of hyposalivation. In addition, this review explores the biological determinants of therapeutic responsiveness, the limitations of current evidence, the interpretation of treatment success beyond salivary flow measurements, and future directions for more individualized and biologically informed PBM strategies.

## Methodology

As a comprehensive narrative review, this article was developed through an interpretative assessment of the available literature concerning PBM and salivary gland dysfunction. Relevant publications were identified through searches of PubMed/MEDLINE, Scopus, and Web of Science, complemented by manual review of reference lists from key articles. The literature selection focused on studies addressing biological mechanisms of salivary dysfunction, photobiomodulation effects, and clinical evidence across different etiologies of hyposalivation and xerostomia.

## Why etiology matters in PBM responsiveness

Although patients with hyposalivation may present with similar clinical complaints, the biological substrate underlying salivary dysfunction may vary substantially between individuals. These biological differences may significantly influence the expected responsiveness to PBM and should be considered before treatment initiation [10, 11]. To better understand how such factors may interfere with therapeutic outcomes, it is first necessary to discuss the biological mechanisms underlying PBM in salivary gland dysfunction.

### PBM mechanisms of action

Following photon absorption by intracellular photoacceptors—particularly within mitochondrial chromophores—PBM promotes increased production of adenosine triphosphate (ATP), enhancing cellular bioenergetics and metabolic activity. This increase in energy availability may favor active transmembrane transport, ionic exchange, protein synthesis, and other cellular processes directly involved in tissue repair and cellular proliferation [10, 13, 14]. Moreover, PBM may directly influence ion channel activity and membrane permeability, facilitating fluid transport and glandular secretion [15].

Beyond its metabolic effects, PBM has also been associated with modulation of inflammatory pathways through reduction of pro-inflammatory mediators and oxidative stress, alongside stimulation of anti-inflammatory and cytoprotective responses. These mechanisms may contribute to preservation of acinar cell viability, improvement of microvascular dynamics, and restoration of tissue integrity following glandular injury [13, 16–18].

In addition, PBM may modulate and reduce oxidative stress by stimulating endogenous antioxidant pathways, including superoxide dismutase (SOD), catalase, and glutathione peroxidase (GPx). Together, these mechanisms may help preserve glandular function, reduce cellular injury, support tissue viability, and promote faster tissue repair and recovery [8, 17, 19–21].

Nevertheless, the magnitude and sustainability of PBM effects are likely dependent on the baseline biological condition of the salivary glands prior to treatment [6, 10, 11, 22]. Consequently, understanding the biological status of glandular tissues before PBM initiation may be essential for interpreting therapeutic outcomes and establishing realistic clinical expectations.

### Determinants of glandular responsiveness to PBM

Since the therapeutic effects of PBM depend on the ability of cells and tissues to respond to photonic stimulation, the presence of residual functional glandular reserve is hypothesized to represent a key factor in determining PBM responsiveness [13, 15]. In this context, functional acinar cells, preserved neural signaling, adequate vascularization, and maintained mitochondrial activity appear likely to contribute to a more favorable response to PBM. Conversely, severely damaged glands with extensive acinar loss, advanced fibrosis, vascular impairment, or marked tissue degeneration may exhibit limited responsiveness due to reduced biological capacity for functional recovery, even in the presence of PBM stimulation [6, 10, 11, 22].

Another important determinant directly related to the preservation of functional glandular reserve is the persistence and chronicity of the etiological insult. Over time, this persistent tissue aggression may contribute to the establishment of a cumulative biological burden, characterized by progressive deterioration of tissue homeostasis and fibrosis, with replacement of functional parenchyma [23]. Consequently, even when photobiomodulatory stimulation is capable of enhancing cellular metabolism and activating tissue repair pathways, reversing cumulative tissue damage—or overcoming an ongoing injurious process—may become substantially more challenging [11].

In addition to chronicity, the magnitude and distribution of harmful stimuli may also directly influence the biological effects achieved through PBM. Past or ongoing insults, acute or gradual exposure, mild or severe aggression, and localized or systemic processes represent important variables capable of substantially influencing glandular status and expected PBM outcomes [24, 25]. Furthermore, in some cases, the multifactorial nature of the injurious stimuli may act synergistically on the biological burden, increasing case complexity and limiting the therapeutic effects of PBM [26, 27]. Additionally, as already well established in the literature, factors such as the patient’s overall health status, water intake, individual physical characteristics, and adequate professional execution of PBM protocols may also play a decisive role in therapeutic success [28–30].

## Etiology-specific perspectives

**Fig. 1:**
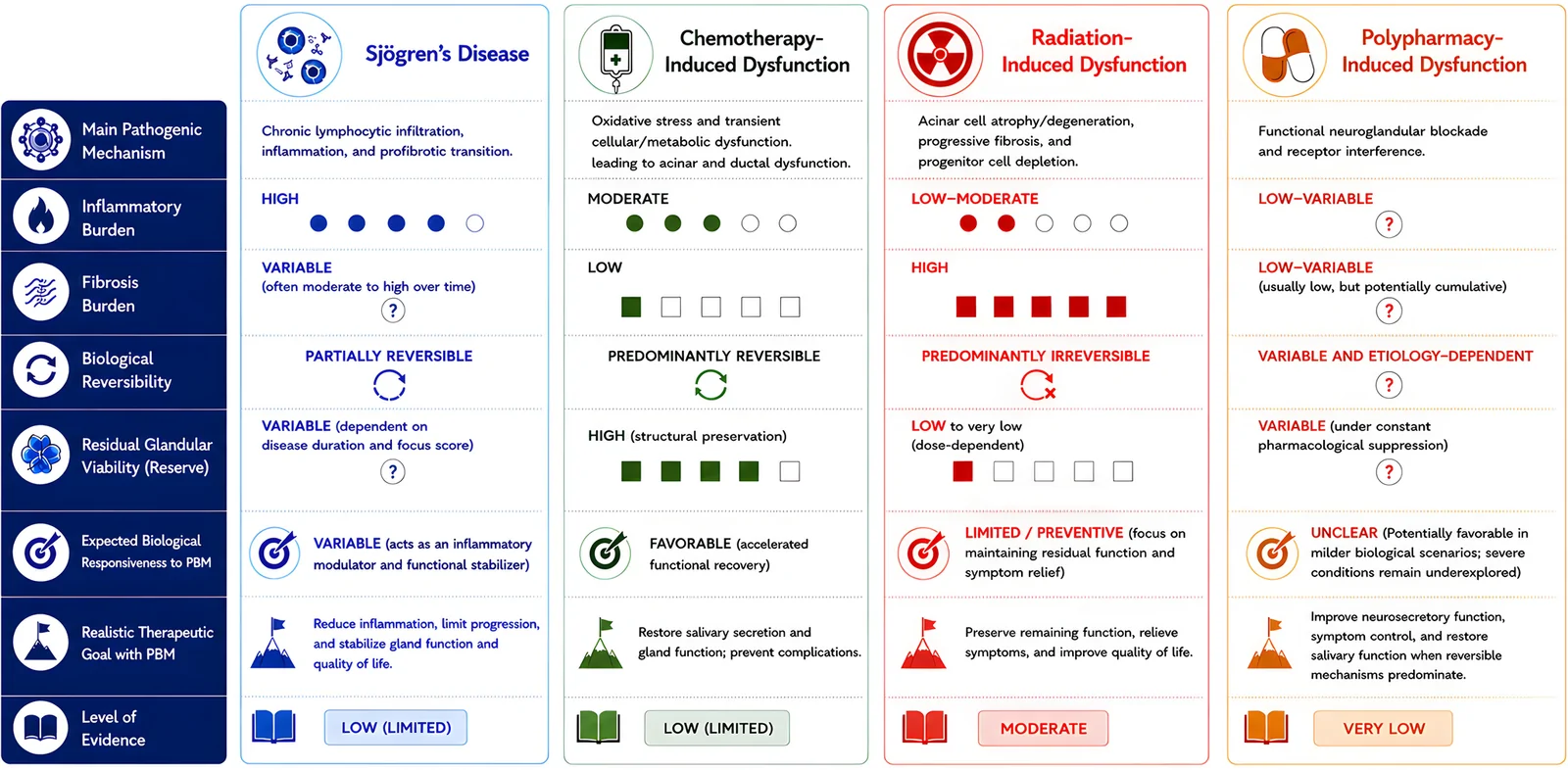
Etiology-dependent determinants of photobiomodulation (PBM) responsiveness in salivary gland dysfunction. This conceptual framework illustrates a hypothesis-generating model of PBM responsiveness based on current biological understanding and available clinical evidence. Distinct etiologies of salivary gland dysfunction may influence PBM responsiveness through differences in pathogenic mechanisms, residual glandular viability, fibrosis, and persistence of the etiological burden

The conditions discussed in the following sections combine the biological determinants previously outlined in different ways, resulting in distinct biological scenarios of salivary dysfunction that may potentially influence therapeutic responsiveness to PBM, as summarized in Fig. 1.

### Sjögren’s disease

Sjögren’s disease (SjD) is an autoimmune rheumatic disease characterized by chronic glandular inflammation resulting from lymphocytic infiltration [31, 32]. It may present as primary SjD, when occurring in isolation, or associated SjD, when associated with other autoimmune connective tissue diseases such as systemic lupus erythematosus, rheumatoid arthritis, or systemic sclerosis [33–36]. Therefore, in addition to the possible biological differences between primary and associated forms of the disease, the influence of the other systemic condition on the patient’s inflammatory and biological profile should also be considered.

Previous studies have demonstrated important similarities between the different forms of SjD regarding clinical, serological, and histopathological features [33, 37–39]. Nevertheless, some distinctions have been described. Primary SjD tends to present with a higher frequency of oral symptoms, parotid enlargement, and increased B-cell activity, evidenced by both greater lymphocytic infiltration and enhanced autoantibody production [33, 38]. In contrast, patients with SjD associated with systemic lupus erythematosus may exhibit more pronounced perivascular lymphocytic infiltration [39], whereas those associated with rheumatoid arthritis frequently present a relatively less symptomatic clinical profile [40].

Beyond chronic inflammatory activity, a subset of patients may progressively develop glandular damage and activation of profibrotic pathways, including mechanisms associated with epithelial–mesenchymal transition (EMT) [41–43]. Under physiological conditions, EMT-related processes participate in tissue repair following acute or moderate injury, contributing to tissue regeneration and structural reorganization [41]. However, under persistent inflammatory conditions such as SjD, sustained activation of these pathways may favor excessive extracellular matrix deposition and progressive glandular fibrosis, ultimately leading to increasingly irreversible functional impairment [41–43].

Recently, it has been demonstrated that, in addition to age — recognized as an important predictor of glandular fibrosis extent — the focal lymphocytic infiltration score in minor salivary gland biopsies from patients with primary SjD also correlates positively with the degree of fibrosis. Furthermore, inclusion of the focus score in regression models based solely on age significantly improves their predictive performance. These findings suggest that higher focus scores may reflect not only greater inflammatory activity, but may also be associated with a biological condition potentially less responsive to functional recovery [44].

It has been widely described that the glandular inflammatory infiltrate observed in SjD is associated with persistent activation of interferon-related pathways, promoting an inflammatory profile resembling that observed during viral infections [32, 45]. In this context, several molecular pathways reportedly modulated by PBM appear to overlap with mechanisms directly implicated in SjD pathophysiology. Some examples are the chronic activation of NF-κB-related inflammatory pathways, excessive production of reactive oxygen species, and impaired antioxidant response [13, 18, 20, 46–50].

### Chemotherapy-induced hyposalivation

Approximately 40% of individuals undergoing chemotherapy (CT) report complaints of dry mouth [9]. In these patients, oxidative stress appears to be one of the main mechanisms underlying these symptoms, potentially triggering inflammatory processes that lead to transient cellular dysfunction affecting the salivary glands. Several antineoplastic agents, particularly 5-fluorouracil and cisplatin, may increase reactive oxygen species (ROS) production and inflammatory cytokine release [17, 51, 52]. This oxidative imbalance may further compromise cellular viability and important signaling pathways involved in acinar secretion, including cholinergic signaling and aquaporin-mediated water transport [53, 54]. Ultimately, these processes may result in salivary hypofunction and xerostomia during treatment [17, 51, 53–55].

Despite the impairment caused during treatment, CT damage is usually predominantly functional rather than permanently structural. As a result, CT-related salivary dysfunction is often transient, with partial or substantial recovery occurring within months after treatment completion, depending on the chemotherapeutic regimen, cumulative dose, and individual patient susceptibility [2, 21, 52, 54]. Collectively, these findings suggest that CT-induced salivary dysfunction appears to frequently develop in glands that remain structurally viable despite presenting significant metabolic and secretory impairment [17, 52, 54, 55].

In this scenario, PBM has become a promising supportive approach, especially considering its therapeutic effects on modulation of oxidative stress and restoration of cellular bioenergetics [14, 20, 55, 56]. Therefore, unlike conditions predominantly characterized by irreversible fibrosis or extensive acinar depletion, CT-related dysfunction may often preserve a biologically responsive substrate potentially capable of functional recovery following photobiomodulatory stimulation [17].

Nevertheless, this potential responsiveness should not be interpreted as universally predictable. The severity of salivary dysfunction may vary according to the chemotherapeutic agent, cumulative dose, and treatment protocol. Likewise, PBM outcomes may depend on the cumulative biological burden established throughout treatment.

### Radiation-induced hyposalivation

Radiation-induced hyposalivation is one of the most severe complications of head and neck radiotherapy (RT) and is frequently associated with long-term glandular dysfunction and xerostomia. Unlike CT-related dysfunction, radiation damage affects around 90% of the patients undergoing head and neck RT [57] and is usually associated with permanent structural alterations, including acinar cell loss, vascular impairment, fibrosis, neural injury, and progressive depletion of salivary progenitor cells [23, 58].

The biological effects of ionizing radiation are strongly linked to excessive reactive oxygen species (ROS) production and oxidative tissue injury [23, 58, 59]. This oxidative and inflammatory environment promotes apoptosis, endothelial injury, tissue degeneration, and progressive loss of glandular function. Serous acinar cells appear to be particularly radiosensitive, which helps explain the marked reduction in salivary secretion commonly observed after head and neck RT [23, 59, 60]. Besides direct epithelial toxicity, radiation also compromises microvascular integrity and neural regulation of salivary glands, impairing tissue oxygenation, nutrient supply, and autonomic stimulation. Over time, fibrosis and depletion of progenitor cells (including c-Kit⁺ and basal epithelial cells) further limit tissue regeneration and spontaneous functional recovery [23, 59, 60].

Oxidative stress also interferes with intracellular calcium signaling pathways involved in salivary secretion, particularly through TRPM2 activation and alterations in STIM1-mediated calcium entry. These alterations impair acinar secretory response and contribute to reduced saliva production after irradiation [23, 59, 61]. As already reported in CT-induced hyposalivation and in SjD, alterations involving aquaporin-5 (AQP5), an important water channel involved in salivary fluid transport, have also been associated with glandular dysfunction after RT. Reduced parasympathetic stimulation and oxidative injury appear to contribute to impaired AQP5 expression and salivary secretion [55, 62–64]. As a consequence of these cumulative alterations, RT also alters salivary composition, resulting in viscous and acidic saliva due to reduced serous secretion and impaired bicarbonate buffering capacity [23, 65–68].

The severity of salivary dysfunction is strongly influenced by total radiation dose, irradiated glandular volume, and RT modality [23, 69–71]. Conventional RT techniques are generally associated with greater salivary gland exposure and higher risk of permanent xerostomia. Studies have shown that mean parotid doses above approximately 26 Gy have been consistently associated with marked and frequently irreversible reductions in SFR [23, 70]. Studies have shown that approximately 80% of patients still experience xerostomia symptoms one year after treatment completion, while nearly 50% remain symptomatic after three years [72, 73]. In some cases, xerostomia may persist or even progressively worsen more than five years after RT [74].

Salivary dysfunction may begin within the first days of RT, even before extensive structural degeneration becomes evident. SFRs may decline by nearly 50% after approximately 10 Gy, suggesting that early functional and neurosecretory alterations play an important role in the development of glandular hypofunction. As treatment progresses, SFR may decrease to less than 20% of baseline levels following a full course of conventional RT (> 60 Gy), frequently resulting in severe and often near-complete functional impairment [23, 70].

More conformal approaches, particularly intensity-modulated radiation therapy (IMRT), have demonstrated improved preservation of salivary gland function by reducing unnecessary radiation exposure to healthy tissues [70, 75]. Similar tissue-sparing effects have also been reported with newer techniques such as volumetric modulated arc therapy (VMAT) and proton therapy [76, 77]. However, even with sparing techniques, the radiation-induced damage may remain substantial [78].

Within this context, based on currently available evidence, PBM appears to act mainly by stimulating residual viable glandular cells rather than fully restoring extensively damaged tissue. Because a substantial portion of glandular tissue may already be irreversibly compromised by fibrosis and acinar cell loss, it may be hypothesized that PBM responsiveness is more limited than that observed in CT-related dysfunction [19, 79]. Even so, clinically relevant benefits may still occur, particularly regarding xerostomia symptoms, oral lubrication, and preservation of residual salivary activity [7, 11, 64, 80, 81]. However, interpretation of these findings should be cautious, as direct comparative clinical evidence remains limited and substantial heterogeneity exists regarding radiation exposure, glandular preservation, and PBM treatment protocols.

Furthermore, preventive PBM protocols initiated during RT appear to provide greater benefit than delayed interventions after treatment completion. Early modulation of oxidative stress and inflammatory response may help preserve residual glandular tissue before extensive fibrosis becomes established. In this context, PBM may also present particular relevance as a preventive or early-intervention strategy [7, 82].

### Polypharmacy-associated hyposalivation

Polypharmacy-associated hyposalivation represents a biologically complex scenario of salivary dysfunction due to the persistent and multifactorial nature of the etiological burden. Unlike conditions characterized by direct inflammatory or structural glandular injury, medication-induced salivary dysfunction initially occurs predominantly as a pharmacologically mediated adverse effect resulting from interference with neuroglandular signaling pathways responsible for salivary secretion. Rather than extensive primary glandular destruction, many xerogenic medications impair autonomic regulation, muscarinic receptor-mediated signaling, ion transport, and fluid secretion mechanisms within acinar cells, leading to functional suppression of SFR despite initial relative preservation of glandular architecture [83–87].

Polypharmacy is particularly relevant because salivary dysfunction rarely results from the action of a single medication alone. Instead, the cumulative anticholinergic burden imposed by multiple drugs — including antidepressants, antihypertensives, antipsychotics, anxiolytics, and diuretics — may simultaneously affect salivary function through distinct and overlapping biological mechanisms [83, 85, 86, 88–91]. In many cases, these medications act both centrally, through reduction of autonomic outflow, and peripherally, by interfering with muscarinic receptor signaling and ion transport in salivary acinar cells. Since parotid glands are highly dependent on parasympathetic stimulation, stimulated SFR appears particularly susceptible to chronic pharmacological inhibition [83, 84, 87].

Physiologically, drug-induced salivary dysfunction predominantly reflects impairment of fluid secretion rather than complete suppression of protein synthesis, resulting in the production of more viscous saliva frequently perceived by patients as oral dryness. Additionally, xerogenic medications may alter salivary composition, buffering capacity, bicarbonate secretion, sodium transport, and salivary pH, compromising oral homeostasis and increasing susceptibility to mucosal injury, dental caries, and opportunistic infections [84, 86, 92, 93]. Although these alterations are initially functional and potentially reversible, prolonged pharmacological exposure may progressively contribute to oxidative imbalance, metabolic stress, persistent salivary hypofunction, and gradual decline of functional glandular reserve [83, 85, 92, 94, 95].

From a translational perspective, polypharmacy-associated hyposalivation presents a particularly challenging therapeutic scenario because, in many cases, medication discontinuation or substitution is clinically impractical. Patients frequently require chronic pharmacological management for systemic diseases and may already have undergone multiple therapeutic adjustments before stabilization of their medical regimen [26, 88, 94]. Consequently, even when PBM is capable of improving mitochondrial metabolism, oxidative balance, tissue repair, and residual glandular function, salivary glands often remain continuously exposed to the etiological insult throughout treatment [13, 94, 96, 97].

This complexity becomes even more pronounced in medically compromised and aging individuals, in whom polypharmacy frequently coexists with chronic systemic conditions capable of independently affecting salivary gland function [86, 88, 94, 95]. Patients undergoing hemodialysis, for example, may simultaneously present systemic inflammation, oxidative stress, autonomic dysfunction, metabolic imbalance, fluid restriction, and extensive pharmacological exposure, all of which may synergistically contribute to salivary hypofunction [86, 98–100].

Similar multifactorial patterns may also be observed in patients with diabetes mellitus, cardiovascular diseases, and multimorbidity-associated frailty, where chronic systemic dysregulation, reduced regenerative capacity, and cumulative pharmacological burden frequently overlap. In these scenarios, salivary dysfunction often reflects not a single dominant etiological mechanism, but rather the progressive accumulation and interaction of multiple biological stressors over time [85, 86, 90, 91, 101, 102], potentially contributing to greater variability and unpredictability in PBM responsiveness. In this context, despite the currently limited and heterogeneous body of evidence, PBM may still provide important symptomatic and functional benefits [11, 97, 100, 103].

Although this review focused on selected etiologies that are particularly relevant to the current PBM literature, salivary gland dysfunction may also occur in several other clinical contexts, including chronic graft-versus-host disease (GVHD), neurological disorders, and other systemic conditions. These scenarios may involve distinct combinations of metabolic, inflammatory, neurosecretory, vascular, and degenerative mechanisms capable of influencing glandular function and potentially affecting responsiveness to PBM. However, the currently available evidence for these conditions remains scarce, precluding a more detailed analysis of these conditions [5, 86].

## Beyond salivary flow: rethinking treatment success

The relationship between xerostomia symptoms and measured SFR is often heterogeneous and only partially correlated. Patients with similar SFR values may present profoundly different symptom perception. This variability likely reflects the complex interaction between salivary quantity, salivary composition, mucosal lubrication, glandular distribution, sensory perception and individual adaptive capacity [12, 104, 105].

Salivary composition, in particular, may be as clinically relevant as salivary volume itself. Distinct etiologies may differentially affect electrolyte balance, buffering capacity, protein secretion, pH, antimicrobial activity, and saliva viscosity, resulting in distinct functional consequences despite apparently comparable SFRs [51, 54, 86, 104, 106–110]. Mucins, in particular, appear to play an important role in the subjective perception of oral dryness due to their essential contribution to saliva rheological properties and formation of the protective salivary film covering oral tissues [111–113]. Alterations in mucin concentration, glycosylation patterns, viscoelasticity, and salivary coating capacity have been reported in xerostomic patients from different etiologies, including Sjögren’s disease, radiation-induced dysfunction, and diabetes-associated hyposalivation [102, 108, 112–114].

Within this framework, minor salivary glands may contribute substantially to symptomatic relief and oral comfort. Although these glands produce a relatively small proportion of total salivary volume, their secretions are particularly rich in mucins and glycoproteins that play essential roles in mucosal lubrication, epithelial protection, swallowing, speech, and maintenance of oral surface integrity [105, 109, 115]. Therefore, despite being frequently overlooked in most PBM protocols reported in the literature [6, 9], minor salivary glands may exert a substantial impact on xerostomia improvement, potentially explaining why some patients report clinically meaningful benefits despite limited objective changes.

Given their potential contribution to xerostomia improvement, appropriate methods for assessing minor salivary glands functional recovery deserve consideration. Although no universally accepted standard currently exists, several approaches have been described, including direct measurement of minor salivary gland secretion rates, clinical assessment of mucosal wetness, imaging-based evaluation of glandular structure and function, and patient-reported xerostomia outcomes. Because minor salivary glands contribute predominantly to mucosal lubrication rather than total salivary volume, subjective symptom improvement and local mucosal hydration parameters may provide clinically meaningful information that is not fully captured by conventional whole-saliva flow measurements [116, 117].

From a translational perspective, treatment goals may differ substantially according to the underlying etiology and biological stage of salivary dysfunction. In conditions predominantly characterized by reversible metabolic or inflammatory dysfunction, partial restoration of glandular secretion may be achievable [8, 17, 118–121]. Conversely, in chronic fibrotic or progressive conditions, therapeutic success may involve stabilization of glandular deterioration, reduction of symptom burden, preservation of residual glandular function, or improvement in oral quality of life rather than complete functional normalization [11, 80, 81].

Importantly, absence of complete SFR normalization should not necessarily be interpreted as therapeutic failure. Interpreting PBM outcomes exclusively through quantitative salivary measurements may oversimplify the biological complexity of salivary dysfunction and underestimate relevant therapeutic benefits [11, 80, 81]. In many chronic or progressive conditions, clinically meaningful benefits may include improved oral comfort, reduction in xerostomia-related distress, decreased oral burning sensation, easier swallowing and speech, reduced dependence on frequent water intake, better sleep quality, and enhanced dietary tolerance [2, 12]. Patients frequently seek treatment hoping for complete restoration of salivary function. Nevertheless, it is the clinician’s responsibility to establish realistic therapeutic expectations, even before initiating PBM.

Longitudinal reassessment also plays a particularly important role during PBM management. Since salivary dysfunction may evolve dynamically according to disease progression, aging, systemic conditions, medication exposure, inflammatory activity, or cumulative tissue damage, periodic reevaluation may help identify changes in biological status and therapeutic responsiveness throughout follow-up. In some patients, maintenance approaches or protocol adjustments may become necessary over time [1, 86, 122].

Comprehensive reassessment should ideally combine both objective and subjective parameters. In addition to unstimulated and stimulated SFR measurements, clinicians may consider validated xerostomia questionnaires, oral health-related quality of life instruments, detailed clinical examination of the oral mucosa, investigation of coexisting etiologies, and complementary laboratory or imaging tests when clinically indicated. In selected cases, autoimmune serology, salivary gland ultrasonography, or evaluation of glandular structural alterations may provide additional information regarding disease activity, residual glandular viability, and potential responsiveness to treatment [12, 24, 71].

Ultimately, individualized reassessment strategies may contribute not only to optimization of PBM protocols, but also to earlier identification of biologically less responsive scenarios, more appropriate therapeutic endpoint selection, and improved long-term clinical management of patients with hyposalivation [11].

## PBM current evidence across different etiologies

Despite the growing interest in PBM as a therapeutic strategy for salivary gland dysfunction, the current literature remains heterogeneous. In this context, synthesizing the existing evidence remains highly relevant not only to critically interpret current findings, but also to identify important methodological and biological gaps that may help guide the development of more robust future research designs.

The current PBM literature is highly unbalanced across etiologies, with most available studies concentrated in radiation-induced salivary dysfunction. Experimental evidence demonstrated that PBM could mitigate radiation-induced salivary gland injury through partial preservation of glandular histoarchitecture and acinar morphology [123]. Meanwhile, several randomized clinical trials and recent systematic reviews have investigated the effects of PBM in patients with head and neck cancer undergoing RT. Importantly, the certainty of evidence remains predominantly low to very low due to small sample sizes, methodological heterogeneity, and high risk of bias across many studies [124].

Overall, the available clinical evidence suggests that PBM may attenuate radiation-induced salivary gland hypofunction, especially when applied in concomitant/prophylactic protocols. Louzeiro et al. [124], in a systematic review, found significant improvements in patients undergoing head and neck RT following PBM, including an overall mean increase of approximately 0.20 mL/min in unstimulated SFR and 0.27 mL/min in stimulated SFR. In contrast, improvements in subjective xerostomia scores have been less consistent across studies [124]. Nevertheless, considering evidence from individual clinical studies not included in the review, the potential symptomatic benefits of PBM for xerostomia should not be disregarded [7, 80, 124–128].

Clinical evidence has also demonstrated potential benefits of PBM in patients with already established post-RT hyposalivation and xerostomia. Palma et al. [129], for example, reported improvements in xerostomia-related quality of life, salivary pH, and SFR in head and neck cancer patients evaluated after RT completion [129]. More recently, Lopez-Garzon et al. [120], in a randomized placebo-controlled clinical trial involving patients with chronic RT-induced xerostomia, observed a nearly significant increase in unstimulated SFR following PBM, with some patients recovering normal SFR levels after treatment [120]. Similarly, Alsoghier et al. [130] reported reductions in xerostomia severity and trends toward increased SFR after PBM therapy in irradiated head and neck cancer patients [130].

Compared with RT-induced salivary dysfunction, evidence evaluating CT-associated hyposalivation is substantially less available, and often not specific [82, 131, 132]. Nevertheless, preliminary studies have shown positive results. In an experimental model, Campos et al. [17], demonstrated promising protective effects of PBM against CT-induced salivary gland damage, particularly regarding biochemical and enzymatic parameters [17]. More recently, Melo et al. (2026) additionally demonstrated that PBM, in association with clarified açaí supplementation, preserved parotid gland morphology, acinar organization, stromal architecture, collagen content, and oxidative biochemical parameters in rats after 5-fluorouracil administration [133]. Furthermore, Arbabi-Kalati et al. [121] and Khalil et al. [134] reported reduced xerostomia severity in patients receiving prophylactic PBM during chemotherapy [121, 134].

In a similar way to CT, evidence regarding PBM for SjD-associated hyposalivation and xerostomia also remains limited. Initial evidence was mainly derived from case reports, including the study by Simões et al. [16], which reported improvement in xerostomia symptoms, salivary gland pain and swelling, and quality of life following prolonged PBM therapy in a patient with SjD [135]. More recently, additional case reports also demonstrated increases in SFR and reductions in xerostomia severity after PBM therapy in patients with SjD [118, 119]. Clinical trials, however, remain scarce and heterogeneous. Cafaro et al. [136], in a pilot placebo-controlled study using laser acupuncture, observed significantly increased salivary production in SjD patients after treatment and during follow-up [136]. Conversely, Fidelix et al. [137], in a randomized clinical trial, did not observe significant improvements in xerostomia scores or SFRs following PBM therapy [137]. These findings illustrate the variability currently observed in the literature and suggest that patient-specific factors, including disease stage, degree of glandular damage, and residual functional reserve, may influence therapeutic responsiveness.

Polypharmacy also represents a poorly explored field for PBM in hyposalivation. Multiple clinical contexts add considerable complexity to this topic, involving different medication combinations, systemic conditions, and therapeutic regimens. Although a few studies have investigated PBM in patients with xerostomia associated with medication use, the literature still lacks studies specifically evaluating the multidrug regimens commonly observed in clinical practice, with currently available evidence being largely restricted to a few isolated pharmacological classes [11, 97, 100, 103, 138].

Consequently, the currently available evidence does not adequately reflect the heterogeneity of polypharmacy-associated salivary dysfunction, limiting conclusions regarding the true therapeutic potential of PBM in this population, particularly in clinically complex cases involving multidrug regimens with high anticholinergic burden. In these patients, unstimulated SFR may become profoundly reduced, sometimes approaching nearly undetectable levels during sialometric evaluation [87, 90, 91]. Importantly, to the best of our knowledge, no clinical studies have specifically evaluated PBM in patients with severe polypharmacy-associated xerostomia characterized by high cumulative anticholinergic exposure.

The heterogeneous and sometimes inconclusive findings currently observed in the PBM literature should not necessarily be interpreted as evidence of therapeutic inefficacy. As discussed throughout this review, substantial biological variability may exist not only between different etiologies, but also among patients within the same etiological category. This variability may directly influence tissue responsiveness to PBM and contribute to the heterogeneity of reported clinical outcomes. Importantly, despite current limitations and inconsistencies, several studies across different etiologies have demonstrated promising functional and symptomatic benefits, reinforcing the therapeutic potential of PBM in the management of salivary gland dysfunction.

An additional challenge when interpreting the current literature relates to the considerable variability in PBM protocols. Differences in wavelength, energy density, irradiation sites, treatment frequency, total delivered dose, and timing of intervention may substantially influence biological responses and clinical outcomes. This methodological heterogeneity complicates direct comparisons across studies and may contribute to the inconsistent findings reported in some clinical settings.

## Future directions

Despite the growing body of evidence supporting PBM for salivary dysfunction, substantial gaps remain regarding the biological determinants of therapeutic responsiveness, optimal treatment protocols, and long-term management strategies across different etiologies. In this context, future investigations should increasingly focus on biologically stratified and etiology-oriented PBM approaches rather than assuming a universally homogeneous therapeutic response. It is essential that future approaches aim to identify which patients and biological scenarios may benefit most from PBM. The identification of predictive biomarkers and imaging parameters associated with PBM responsiveness may also represent an important step toward more individualized therapeutic decision-making.

Another important direction involves expanding outcome assessment beyond SFRs alone. Future studies should incorporate broader functional and patient-centered parameters, aiming to better capture the clinically relevant impact of salivary gland dysfunction and its treatment. Likewise, greater attention should be directed toward the role of minor salivary glands and the potential contribution of intraoral PBM protocols [118, 119, 132], particularly considering the apparent dissociation frequently observed between objective SFR measurements and subjective symptomatic improvement.

The generation of higher-quality evidence through well-designed clinical studies, together with more transparent reporting of PBM parameters and outcomes, should also be a priority for future investigations. Greater methodological consistency in study design and reporting would facilitate evidence synthesis, improve comparability across investigations, and help identify the biological and clinical factors associated with treatment responsiveness. As the evidence base matures, these advances may support the development of more robust, evidence-based clinical guidelines tailored to specific etiologies and clinical scenarios rather than universally uniform treatment protocols.

Importantly, future investigations should also explore the potential synergistic effects between PBM and adjunctive therapeutic strategies. Depending on the biological scenario, combined approaches involving sialogogues [139, 140] preventive radioprotective agents [7, 141–143] medication adjustment [83, 89] anti-inflammatory management [101, 144] acupuncture [145], electroacupuncture [55] or supportive salivary substitutes [1, 139, 146] may provide more comprehensive management of salivary dysfunction than isolated interventions alone. In addition, artificial intelligence and predictive modeling approaches may further support individualized treatment planning by integrating clinical characteristics, imaging findings, salivary biomarkers, and treatment-related variables. Such models could potentially assist in identifying patients who are more likely to respond to PBM, facilitating risk stratification, optimization of treatment protocols, and more personalized therapeutic decision-making [147].

Finally, advances in regenerative medicine, salivary gland engineering, mitochondrial modulation, stem cell-based therapies, and personalized biological profiling may progressively reshape the future management of hyposalivation [148–153]. Within this evolving landscape, PBM will likely continue to occupy an important role not as a universally curative intervention, but as part of increasingly individualized and biologically integrated therapeutic strategies adapted to the specific pathophysiological context of each patient.

The present article is an interpretative, hypothesis-generating synthesis of current biological and clinical evidence and should be interpreted within the methodological limitations inherent to narrative reviews. Factors such as publication bias, the heterogeneity of the available literature, and the uneven distribution of evidence across different etiologies may influence the concepts discussed. Therefore, the proposed etiology-dependent model should be viewed mainly as a conceptual framework intended to guide future research and clinical reasoning.

Increasing recognition of the biological heterogeneity underlying hyposalivation reinforces the importance of personalized medicine strategies. Ultimately, future PBM strategies will likely depend less on universally standardized protocols and more on biologically individualized approaches capable of adapting treatment parameters, therapeutic combinations, and clinical expectations according to the specific pathophysiological context of each patient.

## Conclusion

The biological heterogeneity underlying salivary gland dysfunction suggests that PBM responsiveness should not be interpreted as universally predictable. Instead, therapeutic outcomes likely depend on the reversibility of glandular injury, residual functional viability, and persistence of the etiological burden. Importantly, therapeutic success should not be interpreted exclusively through SFR measurements, since clinically relevant benefits may occur even in the absence of complete SFR normalization, particularly through symptom reduction, preservation of residual function, and improvement of oral quality of life. Therefore, future PBM strategies will likely benefit from more individualized and biologically informed approaches aligned with the specific pathogenic context of each patient. Recognizing etiological heterogeneity may also help reposition PBM not as a universally curative therapy, but as a biologically adaptive strategy integrated into personalized management of salivary gland dysfunction.

## Data Availability

No datasets were generated or analysed during the current study.

## Declarations

### AI Statement

During manuscript preparation, the authors used OpenAI’s ChatGPT (GPT-5) to assist with readability and refinement of graphical elements used in figures. All content was critically reviewed and approved by the authors, who assume full responsibility for the final manuscript.

## References

[1] Villa A, Connell C, Abati S (2014) Diagnosis and management of xerostomia and hyposalivation. Ther Clin Risk Manag. 10.2147/TCRM.S76282 25653532 10.2147/TCRM.S76282 PMC4278738

[2] Jensen SB, Pedersen AML, Vissink A et al (2010) A systematic review of salivary gland hypofunction and xerostomia induced by cancer therapies: prevalence, severity and impact on quality of life. Support Care Cancer 18:1039–1060. 10.1007/s00520-010-0827-8 20237805 10.1007/s00520-010-0827-8

[3] Diaz-Arnold AM, Marek CA (2002) The impact of saliva on patient care: A literature review. J Prosthet Dent 88:337–343. 10.1067/mpr.2002.128176 12426506 10.1067/mpr.2002.128176

[4] Lin A, Helgeson ES, Treister NS et al (2022) The impact of head and neck radiotherapy on salivary flow and quality of life: results of the ORARAD study. Oral Oncol 127:105783. 10.1016/j.oraloncology.2022.105783 35231809 10.1016/j.oraloncology.2022.105783 PMC8977268

[5] Turner MD (2016) Hyposalivation and Xerostomia. Dent Clin North Am 60:435–443. 10.1016/j.cden.2015.11.003 27040294 10.1016/j.cden.2015.11.003

[6] Golež A, Frangež I, Cankar K et al (2022) Effects of low-level light therapy on xerostomia related to hyposalivation: a systematic review and meta-analysis of clinical trials. Lasers Med Sci 37:745–758. 10.1007/s10103-021-03392-0 34409539 10.1007/s10103-021-03392-0

[7] Simões A, de Campos L, de Souza DN et al (2010) Laser phototherapy as topical prophylaxis against radiation-induced xerostomia. Photomed Laser Surg 28:357–363. 10.1089/pho.2009.2486 19814701 10.1089/pho.2009.2486

[8] Campos L, Magliano GC, Hotsumi AM et al (2023) Photobiomodulation therapy mitigates salivary gland damage induced by radioactive iodine ablation. Photonics (Basel) 10:611. 10.3390/photonics10060611

[9] Oliveira SV, Batista JVF, Gutierres GG et al (2024) The supportive use of photobiomodulation on salivary glands: a narrative review and meta-analysis. Eur Arch Otorhinolaryngol 281:2793–2805. 10.1007/s00405-023-08425-8 38189964 10.1007/s00405-023-08425-8

[10] Arany PR (2025) Photobiomodulation therapy. JADA Found Sci 4:100045. 10.1016/j.jfscie.2025.100045 42238110 10.1016/j.jfscie.2025.100045 PMC13229060

[11] Ferrandez-Pujante A, Pons-Fuster E, López-Jornet P (2022) Efficacy of Photobiomodulation in Reducing Symptomatology and Improving the Quality of Life in Patients with Xerostomia and Hyposalivation: A Randomized Controlled Trial. J Clin Med 11:3414. 10.3390/jcm11123414 35743485 10.3390/jcm11123414 PMC9225194

[12] EISBRUCH A, RHODUS N, ROSENTHAL D et al (2003) How should we measure and report radiotherapy-induced xerostomia? Semin Radiat Oncol 13:226–234. 10.1016/S1053-4296(03)00033-X 12903012 10.1016/S1053-4296(03)00033-X

[13] Bjordal JM, Lopes-Martins RAB, Frigo L (2015) Low level laser therapy – mechanism of action. Lasers in Dentistry. Wiley, pp 27–33

[14] Karu TI (2015) Cellular mechanisms of photobiomodulation. Lasers in Dentistry. Wiley, pp 23–26

[15] Sharma SK, Sardana S, Hamblin MR (2023) Role of opsins and light or heat activated transient receptor potential ion channels in the mechanisms of photobiomodulation and infrared therapy. J Photochem Photobiol 13:100160. 10.1016/j.jpap.2023.100160

[16] Simões A, Siqueira WL, Lamers ML et al (2009) Laser phototherapy effect on protein metabolism parameters of rat salivary glands. Lasers Med Sci 24:202–208. 10.1007/s10103-008-0548-0 18309457 10.1007/s10103-008-0548-0

[17] Campos L, Nicolau J, Arana-Chavez VE, Simões A (2014) Effect of Laser Phototherapy on Enzymatic Activity of Salivary Glands of Hamsters Treated with 5‐Fluorouracil. Photochem Photobiol 90:667–672. 10.1111/php.12195 24172058 10.1111/php.12195

[18] Cotomacio CC, Calarga CC, Yshikawa BK et al (2021) Wound healing process with different photobiomodulation therapy protocols to treat 5-FU-induced oral mucositis in hamsters. Arch Oral Biol 131:105250. 10.1016/j.archoralbio.2021.105250 34482219 10.1016/j.archoralbio.2021.105250

[19] de Freitas LF, Hamblin MR (2016) Proposed mechanisms of photobiomodulation or low-level light therapy. IEEE J Sel Top Quantum Electron 22:348–364. 10.1109/JSTQE.2016.2561201 10.1109/JSTQE.2016.2561201 PMC5215870 28070154

[20] Hamblin MR (2018) Mechanisms and Mitochondrial Redox Signaling in Photobiomodulation. Photochem Photobiol 94:199–212. 10.1111/php.12864 29164625 10.1111/php.12864 PMC5844808

[21] Nguyen H, Sangha S, Pan M et al (2022) Oxidative stress and chemoradiation-induced oral mucositis: a scoping review of in vitro, in vivo and clinical studies. Int J Mol Sci 23:4863. 10.3390/ijms23094863 35563254 10.3390/ijms23094863 PMC9101413

[22] Saleh J, Figueiredo MAZ, Cherubini K et al (2014) Effect of Low-Level Laser Therapy on Radiotherapy-Induced Hyposalivation and Xerostomia: A Pilot Study. Photomed Laser Surg 32:546–552. 10.1089/pho.2014.3741 25302460 10.1089/pho.2014.3741

[23] Jasmer KJ, Gilman KE, Muñoz Forti K et al (2020) Radiation-induced salivary gland dysfunction: mechanisms, therapeutics and future directions. J Clin Med 9:4095. 10.3390/jcm9124095 33353023 10.3390/jcm9124095 PMC7767137

[24] Cheng SCH, Wu VWC, Kwong DLW, Ying MTC (2011) Assessment of post-radiotherapy salivary glands. Br J Radiol 84:393–402. 10.1259/bjr/66754762 21511748 10.1259/bjr/66754762 PMC3473647

[25] Dirix P, Nuyts S, Van den Bogaert W (2006) Radiation-induced xerostomia in patients with head and neck cancer. Cancer 107:2525–2534. 10.1002/cncr.22302 17078052 10.1002/cncr.22302

[26] Villa A, Wolff A, Aframian D et al (2015) World workshop on oral medicine VI: a systematic review of medication-induced salivary gland dysfunction: prevalence, diagnosis, and treatment. Clin Oral Investig 19:1563–1580. 10.1007/s00784-015-1488-2 25994331 10.1007/s00784-015-1488-2

[27] Toan NK, Ahn S-G (2021) Aging-related metabolic dysfunction in the salivary gland: a review of the literature. Int J Mol Sci 22:5835. 10.3390/ijms22115835 34072470 10.3390/ijms22115835 PMC8198609

[28] Tunér J, Ribeiro MS, Simões A (2015) Dosimetry. In: Freitas PM, Simões A (eds) Lasers in Dentistry: Guide for Clinical Practice. pp 48–55

[29] de Brito CBT, Calarga CC, Lima FS et al (2025) Photosensitivity episodes related to skin color in people treated with dual-wavelength low power laser therapy: a retrospective cohort study. Photodermatol Photoimmunol Photomed. 10.1111/phpp.70042 40785283 10.1111/phpp.70042 PMC12336633

[30] Correa L, Meirelles L, Carneiro RCG et al (2025) Photobiomodulation in the parotid and submandibular glands: a Monte Carlo simulation of photon penetration using 525-nm, 660-nm, and 850-nm wavelengths. Lasers Med Sci 40:269. 10.1007/s10103-025-04507-7 40498366 10.1007/s10103-025-04507-7

[31] Fox RI (2005) Sjögren’s syndrome. Lancet 366:321–331. 10.1016/S0140-6736(05)66990-5 16039337 10.1016/S0140-6736(05)66990-5

[32] Nocturne G, Mariette X (2013) Advances in understanding the pathogenesis of primary Sjögren’s syndrome. Nat Rev Rheumatol 9:544–556. 10.1038/nrrheum.2013.110 23857130 10.1038/nrrheum.2013.110

[33] Hernández-Molina G, Ávila-Casado C, Cárdenas-Velázquez F et al (2010) Similarities and differences between primary and secondary Sjögren’s syndrome. J Rheumatol 37:800–808. 10.3899/jrheum.090866 20194453 10.3899/jrheum.090866

[34] van Santen JS, Assy Z, Bikker FJ et al (2023) The diagnostic power of salivary electrolytes for Sjögren’s disease: a systematic literature review and meta-analysis. Clin Exp Rheumatol 41:2511–2524. 10.55563/clinexprheumatol/648k4u 38079344 10.55563/clinexprheumatol/648k4u

[35] Muszyński D, Kucharski R, Marek-Trzonkowska N et al (2025) Treatment of xerostomia in Sjögren’s syndrome – what effect does it have on the oral microbiome? Front Cell Infect Microbiol 15:1–8. 10.3389/fcimb.2025.1484951 10.3389/fcimb.2025.1484951 PMC12034647 40297612

[36] Ramos-Casals M, Baer AN, Brito-Zerón M et al (2025) 2023 International Rome consensus for the nomenclature of Sjögren disease. Nat Rev Rheumatol 21:426–437. 10.1038/s41584-025-01268-z 40494962 10.1038/s41584-025-01268-z

[37] Salliot C, Mouthon L, Ardizzone M et al (2006) Sjogren’s syndrome is associated with and not secondary to systemic sclerosis. Rheumatology 46:321–326. 10.1093/rheumatology/kel252 16877466 10.1093/rheumatology/kel252

[38] Kucuk U, Sarioglu S, Cetin P et al (2018) Histopathological differences between primary Sjögren’s syndrome and Sjögren’s syndrome accompanied by scleroderma. Indian J Pathol Microbiol 61:319. 10.4103/IJPM.IJPM_416_17 30004047 10.4103/IJPM.IJPM_416_17

[39] Manoussakis MN, Georgopoulou C, Zintzaras E et al (2004) Sjögren’s syndrome associated with systemic lupus erythematosus: Clinical and laboratory profiles and comparison with primary Sjögren’s syndrome. Arthritis Rheum 50:882–891. 10.1002/art.20093 15022331 10.1002/art.20093

[40] Andonopoulos AP, Drosos AA, Skopouli FN et al (1987) Secondary Sjögren’s syndrome in rheumatoid arthritis. J Rheumatol 14:1098–1103 3437415

[41] Sisto M, Ribatti D, Lisi S (2022) Sjögren’s syndrome-related organs fibrosis: hypotheses and realities. J Clin Med. 10.3390/jcm11123551 35743618 10.3390/jcm11123551 PMC9224630

[42] Sisto M, Lisi S, Ribatti D (2018) The role of the epithelial-to-mesenchymal transition (EMT) in diseases of the salivary glands. Histochem Cell Biol 150:133–147. 10.1007/s00418-018-1680-y 29789993 10.1007/s00418-018-1680-y

[43] Ma D, Feng Y, Lin X (2024) Immune and non-immune mediators in the fibrosis pathogenesis of salivary gland in Sjögren’s syndrome. Front Immunol 15:1421436. 10.3389/fimmu.2024.1421436 39469708 10.3389/fimmu.2024.1421436 PMC11513355

[44] Leehan KM, Pezant NP, Rasmussen A et al (2018) Minor salivary gland fibrosis in Sjögren’s syndrome is elevated, associated with focus score and not solely a consequence of aging. Clin Exp Rheumatol 36 Suppl 112:80–88 PMC5913007 29148407

[45] Yoon J, Lee M, Ali AA et al (2022) Mitochondrial double-stranded RNAs as a pivotal mediator in the pathogenesis of Sjӧgren’s syndrome. Mol Ther Nucleic Acids 30:257–269. 10.1016/j.omtn.2022.09.020 10.1016/j.omtn.2022.09.020 PMC9576540 36284513

[46] Zhu W, Chen L, Li Y et al (2026) Maidong Dishao Decoction can inhibit the activation of the cGAS-STING signaling pathway of Sjögren’s syndrome by reducing oxidative stress in salivary gland epithelial cells. J Ethnopharmacol 354:120470. 10.1016/j.jep.2025.120470 40882790 10.1016/j.jep.2025.120470

[47] Lisi S, Sisto M, Lofrumento DD, D’Amore M (2012) Sjögren’s syndrome autoantibodies provoke changes in gene expression profiles of inflammatory cytokines triggering a pathway involving TACE/NF-κB. Lab Invest 92:615–624. 10.1038/labinvest.2011.190 22157716 10.1038/labinvest.2011.190

[48] Sisto M, Ribatti D, Lisi S (2020) Understanding the complexity of Sjögren’s syndrome: remarkable progress in elucidating NF-κB mechanisms. J Clin Med 9:2821. 10.3390/jcm9092821 32878252 10.3390/jcm9092821 PMC7563658

[49] Li P, Yang Y, Jin Y et al (2019) B7-H3 participates in human salivary gland epithelial cells apoptosis through NF-κB pathway in primary Sjögren’s syndrome. J Transl Med 17:268. 10.1186/s12967-019-2017-x 31412888 10.1186/s12967-019-2017-x PMC6694606

[50] Cotomacio CC, Yshikawa BK, Calarga CC et al (2023) Red, infrared, and simultaneous laser-wavelengths irradiation effects on 5‐fluorouracil‐induced oral mucositis in hamsters. J Biophotonics. 10.1002/jbio.202300156 37420314 10.1002/jbio.202300156

[51] Hosseini M-S, Sanaie S, Mahmoodpoor A et al (2024) Cancer treatment-related xerostomia: basics, therapeutics, and future perspectives. Eur J Med Res 29:571. 10.1186/s40001-024-02167-x 39614391 10.1186/s40001-024-02167-x PMC11607820

[52] Bomfin LE, Braga CM, Oliveira TA et al (2017) 5-Fluorouracil induces inflammation and oxidative stress in the major salivary glands affecting salivary flow and saliva composition. Biochem Pharmacol 145:34–45. 10.1016/j.bcp.2017.08.024 28867645 10.1016/j.bcp.2017.08.024

[53] Golnarnik G, Søland TM, Galtung HK, Haug TM (2024) Hydrogen peroxide disrupts the regulatory pathway of saliva secretion in two salivary acinar rat cell lines. Front Mol Biosci 11. 10.3389/fmolb.2024.1480721 10.3389/fmolb.2024.1480721 PMC11599174 39606036

[54] Silva GER, Padovezi ACE, Barzotti RJ et al (2025) Induction of oxidative stress and functional impairment by Doxorubicin in rat salivary glands. Naunyn Schmiedebergs Arch Pharmacol. 10.1007/s00210-025-04767-8 41152615 10.1007/s00210-025-04767-8

[55] Nguyen T-H, Chiu K-C, Shih Y-H et al (2023) Protective effect of electroacupuncture on chemotherapy-induced salivary gland hypofunction in a mouse model. Int J Mol Sci 24:11654. 10.3390/ijms241411654 37511411 10.3390/ijms241411654 PMC10380826

[56] Rupel K, Zupin L, Colliva A et al (2018) Photobiomodulation at Multiple Wavelengths Differentially Modulates Oxidative Stress In Vitro and In Vivo. Oxid Med Cell Longev 2018:6510159. 10.1155/2018/6510159 30534349 10.1155/2018/6510159 PMC6252186

[57] Chen L, Chen J, Huang Y et al (2026) The Prevalence of Radiation-Induced Xerostomia in Patients with Head and Neck Cancer: A Systematic Review and Meta-analysis. J Maxillofac Oral Surg. 10.1007/s12663-025-02915-441971497

[58] Acauan MD, Figueiredo MAZ, Cherubini K et al (2015) Radiotherapy-induced salivary dysfunction: Structural changes, pathogenetic mechanisms and therapies. Arch Oral Biol 60:1802–1810. 10.1016/j.archoralbio.2015.09.014 26454716 10.1016/j.archoralbio.2015.09.014

[59] Liu Z, Dong L, Zheng Z et al (2021) Mechanism, prevention, and treatment of radiation-induced salivary gland injury related to oxidative stress. Antioxidants (Basel) 10:1666. 10.3390/antiox10111666 34829539 10.3390/antiox10111666 PMC8614677

[60] Konings AWT, Coppes RP, Vissink A (2005) On the mechanism of salivary gland radiosensitivity. Int J Radiat Oncol Biol Phys 62:1187–1194. 10.1016/j.ijrobp.2004.12.051 15990024 10.1016/j.ijrobp.2004.12.051

[61] Ambudkar I (2018) Calcium signaling defects underlying salivary gland dysfunction. Biochimica et Biophysica Acta (BBA) - Molecular Cell Research 1865:1771–1777. 10.1016/j.bbamcr.2018.07.002 30006140 10.1016/j.bbamcr.2018.07.002

[62] Li Z, Zhao D, Gong B et al (2006) Decreased Saliva Secretion and Down-Regulation of AQP5 in Submandibular Gland in Irradiated Rats. Radiat Res 165:678–687. 10.1667/RR3569.1 16802868 10.1667/RR3569.1

[63] Araujo MVT, Spadella MA, Chies AB et al (2018) Effect of low radiation dose on the expression and location of aquaporins in rat submandibular gland. Tissue Cell 53:104–110. 10.1016/j.tice.2018.06.006 30060820 10.1016/j.tice.2018.06.006

[64] Krueger GF, de Oliveira MC, Gassen HT et al (2020) Evaluation of aquaporins 1 and 5 expression in rat parotid glands after volumetric modulated arc radiotherapy and use of low-level laser therapy at different times. J Lasers Med Sci 11:262–267. 10.34172/jlms.2020.44 32802285 10.34172/jlms.2020.44 PMC7369551

[65] Ventura TMO, Ribeiro NR, Taira EA et al (2022) Radiotherapy changes the salivary proteome in head and neck cancer patients: evaluation before, during, and after treatment. Clin Oral Investig 26:225–258. 10.1007/s00784-021-03995-5 34052889 10.1007/s00784-021-03995-5

[66] Vidotto A, Henrique T, Raposo LS et al (2011) Salivary and serum proteomics in head and neck carcinomas: before and after surgery and radiotherapy. Cancer Biomark 8:95–107. 10.3233/CBM-2011-0205 10.3233/CBM-2011-0205 PMC13015853 21896997

[67] Jehmlich N, Stegmaier P, Golatowski C et al (2015) Differences in the whole saliva baseline proteome profile associated with development of oral mucositis in head and neck cancer patients undergoing radiotherapy. J Proteom 125:98–103. 10.1016/j.jprot.2015.04.030 10.1016/j.jprot.2015.04.030 25997676

[68] Hynne H, Aqrawi LA, Jensen JL et al (2022) Proteomic profiling of saliva and tears in radiated head and neck cancer patients as compared to primary Sjögren’s syndrome patients. Int J Mol Sci 23:3714. 10.3390/ijms23073714 35409074 10.3390/ijms23073714 PMC8998953

[69] Jellema AP, Doornaert P, Slotman BJ et al (2005) Does radiation dose to the salivary glands and oral cavity predict patient-rated xerostomia and sticky saliva in head and neck cancer patients treated with curative radiotherapy? Radiother Oncol 77:164–171. 10.1016/j.radonc.2005.10.002 16256229 10.1016/j.radonc.2005.10.002

[70] Nutting CM, Morden JP, Harrington KJ et al (2011) Parotid-sparing intensity modulated versus conventional radiotherapy in head and neck cancer (PARSPORT): a phase 3 multicentre randomised controlled trial. Lancet Oncol 12:127–136. 10.1016/S1470-2045(10)70290-4 21236730 10.1016/S1470-2045(10)70290-4 PMC3033533

[71] Makkonen TA, Nordman E (1987) Estimation of Long-Term Salivary Gland Damage Induced by Radiotherapy. Acta Oncol (Madr) 26:307–312. 10.3109/02841868709089980 10.3109/02841868709089980 3689584

[72] You D, Zhou J, Zhu G et al (2026) Association of irradiation dose and increased incidence of xerostomia inpatients with nasopharyngeal carcinoma. Radiat Med Prot 7:73–80. 10.1016/j.radmp.2026.02.003

[73] Abou-Bakr A, Hassanein FEA, William H, Ibrahim SS (2025) Prevalence of late xerostomia and hyposalivation with associated risk factors in survivors of head and neck cancer after radiotherapy: a multi-centric cross-sectional study. Radiat Oncol 20:162. 10.1186/s13014-025-02737-1 41204325 10.1186/s13014-025-02737-1 PMC12593948

[74] Meßmer M-B, Thomsen A, Kirste S et al (2011) Xerostomia after radiotherapy in the head&neck area: Long-term observations. Radiother Oncol 98:48–50. 10.1016/j.radonc.2010.10.013 21044803 10.1016/j.radonc.2010.10.013

[75] Lambrecht M, Nevens D, Nuyts S (2013) Intensity-modulated radiotherapy vs. parotid-sparing 3D conformal radiotherapy. Strahlenther Onkol 189:223–229. 10.1007/s00066-012-0289-7 23319256 10.1007/s00066-012-0289-7

[76] Kiafi P, Chalkia M, Kouri MA et al (2024) Photon vs proton radiation therapy in head and neck cancer: a review of dosimetric advantages and patient quality of life. J Cancer Metastasis Treat. 10.20517/2394-4722.2024.79

[77] Zhang H, Cao Y, Antone J et al (2019) A Model-based method for assessment of salivary gland and planning target volume dosimetry in volumetric-modulated arc therapy planning on head-and-neck cancer. J Med Phys 44:201. 10.4103/jmp.JMP_19_19 31576068 10.4103/jmp.JMP_19_19 PMC6764180

[78] Randall K, Stevens J, Yepes JF et al (2013) Analysis of factors influencing the development of xerostomia during intensity-modulated radiotherapy. Oral Surg Oral Med Oral Pathol Oral Radiol 115:772–779. 10.1016/j.oooo.2013.01.006 23523462 10.1016/j.oooo.2013.01.006 PMC3665637

[79] Vissink A, Jansma J, Spijkervet FKL et al (2003) Oral sequelae of head and neck radiotherapy. Crit Rev Oral Biol Med 14:199–212. 10.1177/154411130301400305 12799323 10.1177/154411130301400305

[80] Campos L, Simões A, Sá PHRN, Eduardo CDP (2009) Improvement in quality of life of an oncological patient by laser phototherapy. Photomed Laser Surg 27:371–374. 10.1089/pho.2008.2300 18800946 10.1089/pho.2008.2300

[81] Oton-Leite AF, De Corrêa AC, Morais MO et al (2012) Effect of intraoral low-level laser therapy on quality of life of patients with head and neck cancer undergoing radiotherapy. Head Neck 34:398–404. 10.1002/hed.21737 21472883 10.1002/hed.21737

[82] Gonnelli FAS, Palma LF, Giordani AJ et al (2016) Low-level laser therapy for the prevention of low salivary flow rate after radiotherapy and chemotherapy in patients with head and neck cancer. Radiol Bras 49:86–91. 10.1590/0100-3984.2014.0144 27141130 10.1590/0100-3984.2014.0144 PMC4851476

[83] Wolff A, Joshi RK, Ekström J et al (2017) A guide to medications inducing salivary gland dysfunction, xerostomia, and subjective sialorrhea: a systematic review sponsored by the World Workshop on Oral Medicine VI. Drugs R D 17:1–28. 10.1007/s40268-016-0153-9 27853957 10.1007/s40268-016-0153-9 PMC5318321

[84] Proctor GB (2016) The physiology of salivary secretion. Periodontol 2000 70:11–25. 10.1111/prd.12116 26662479 10.1111/prd.12116

[85] Tiisanoja A, Syrjälä A-M, Kullaa A, Ylöstalo P (2020) Anticholinergic burden and dry mouth in middle-aged people. JDR Clin Transl Res 5:62–70. 10.1177/2380084419844511 10.1177/2380084419844511 31013461

[86] Saleh J, Figueiredo MAZ, Cherubini K, Salum FG (2015) Salivary hypofunction: An update on aetiology, diagnosis and therapeutics. Arch Oral Biol 60:242–255. 10.1016/j.archoralbio.2014.10.004 25463902 10.1016/j.archoralbio.2014.10.004

[87] Arany S, Kopycka-Kedzierawski DT, Caprio TV, Watson GE (2021) Anticholinergic medication: Related dry mouth and effects on the salivary glands. Oral Surg Oral Med Oral Pathol Oral Radiol 132:662–670. 10.1016/j.oooo.2021.08.015 34593340 10.1016/j.oooo.2021.08.015 PMC9112430

[88] Sreebny LM, Schwartz SS (1997) A reference guide to drugs and dry mouth – 2nd edition. Gerodontology 14:33–47. 10.1111/j.1741-2358.1997.00033.x 9610301 10.1111/j.1741-2358.1997.00033.x

[89] Thomson WM, Chalmers JM, Spencer AJ, Slade GD (2000) Medication and Dry Mouth: Findings from a Cohort Study of Older People. J Public Health Dent 60:12–20. 10.1111/j.1752-7325.2000.tb03286.x 10734611 10.1111/j.1752-7325.2000.tb03286.x

[90] Ringstad IB, Skovlund E, Roa Santervas L et al (2026) Association Between Anticholinergic Burden and Dry Mouth Severity in Acutely Ill Older Adults. Drugs Aging 43:361–371. 10.1007/s40266-026-01286-w 41824280 10.1007/s40266-026-01286-w PMC13038769

[91] Tan ECK, Lexomboon D, Sandborgh-Englund G et al (2018) Medications That Cause Dry Mouth As an Adverse Effect in Older People: A Systematic Review and Metaanalysis. J Am Geriatr Soc 66:76–84. 10.1111/jgs.15151 29071719 10.1111/jgs.15151

[92] Kim Y-J (2023) Xerostomia and its cellular targets. Int J Mol Sci 24:5358. 10.3390/ijms24065358 36982432 10.3390/ijms24065358 PMC10049126

[93] Mizuhashi F, Morita T, Toya S et al (2020) Protein Ingredient in Saliva on Oral Dryness Patients Caused by Calcium Blocker. Geriatrics 5:70. 10.3390/geriatrics5040070 33036340 10.3390/geriatrics5040070 PMC7709676

[94] Hilmer SN (2007) A Drug Burden Index to Define the Functional Burden of Medications in Older People. Arch Intern Med 167:781. 10.1001/archinte.167.8.781 17452540 10.1001/archinte.167.8.781

[95] Nagler RM (2004) Salivary Glands and the Aging Process: Mechanistic Aspects, Health-Status and Medicinal-Efficacy Monitoring. Biogerontology 5:223–233. 10.1023/B:BGEN.0000038023.36727.50 15314272 10.1023/B:BGEN.0000038023.36727.50

[96] Chavantes MC, Ribeiro MS, Pinto NC (2015) Low power lasers. Lasers in Dentistry. Wiley, pp 19–22

[97] Terlević Dabić D, Jurišić S, Vučićević Boras V et al (2016) The Effectiveness of Low-Level Laser Therapy in Patients with Drug-Induced Hyposalivation: A Pilot Study. Photomed Laser Surg 34:389–393. 10.1089/pho.2016.4109 27415181 10.1089/pho.2016.4109

[98] Sharifnia AM, Chu G, Zhang X et al (2024) Comparative efficacy of non-pharmacological interventions on xerostomia and salivary flow rate among haemodialysis patients: A systematic review and network meta-analysis. Clin Kidney J 17. 10.1093/ckj/sfae334 10.1093/ckj/sfae334 PMC11631358 39664992

[99] Anuradha B, Katta S, Kode V et al (2015) Oral and salivary changes in patients with chronic kidney disease: A clinical and biochemical study. J Indian Soc Periodontol 19:297. 10.4103/0972-124X.154178 26229271 10.4103/0972-124X.154178 PMC4520115

[100] Pavesi VCS, Martins MD, Coracin FL et al (2021) Effects of photobiomodulation in salivary glands of chronic kidney disease patients on hemodialysis. Lasers Med Sci 36:1209–1217. 10.1007/s10103-020-03158-0 33745088 10.1007/s10103-020-03158-0

[101] López-Pintor RM, Casañas E, González-Serrano J et al (2016) Xerostomia, Hyposalivation, and Salivary Flow in Diabetes Patients. J Diabetes Res 2016:1–15. 10.1155/2016/4372852 10.1155/2016/4372852 PMC4958434 27478847

[102] Romero AC, Ibuki FK, Nogueira FN (2012) Sialic acid reduction in the saliva of streptozotocin induced diabetic rats. Arch Oral Biol 57:1189–1193. 10.1016/j.archoralbio.2012.02.016 22421632 10.1016/j.archoralbio.2012.02.016

[103] Varellis MLZ, Bussadori SK, Pavesi VCS et al (2024) Evaluation of photobiomodulation in the salivary production of patients with hyposalivation induced by antihypertensive drugs – a blind, randomized, controlled clinical trial. Complement Ther Clin Pract 56:101845. 10.1016/j.ctcp.2024.101845 38608541 10.1016/j.ctcp.2024.101845

[104] Dawes C (2004) How Much Saliva Is Enough for Avoidance of Xerostomia? Caries Res 38:236–240. 10.1159/000077760 15153694 10.1159/000077760

[105] Dawes C, Pedersen AML, Villa A et al (2015) The functions of human saliva: A review sponsored by the World Workshop on Oral Medicine VI. Arch Oral Biol 60:863–874. 10.1016/j.archoralbio.2015.03.004 25841068 10.1016/j.archoralbio.2015.03.004

[106] Pramanik R, Osailan SM, Challacombe SJ et al (2010) Protein and mucin retention on oral mucosal surfaces in dry mouth patients. Eur J Oral Sci 118:245–253. 10.1111/j.1600-0722.2010.00728.x 20572857 10.1111/j.1600-0722.2010.00728.x

[107] Fusconi M, Candelori F, Weiss L et al (2021) Qualitative mucin disorders in patients with primary Sjögren’s syndrome: a literature review. Med Oral Patol Oral Cir Bucal. 10.4317/medoral.23996 33247578 10.4317/medoral.23996 PMC7806352

[108] Imanguli MM, Atkinson JC, Harvey KE et al (2007) Changes in salivary proteome following allogeneic hematopoietic stem cell transplantation. Exp Hematol 35:184–192. 10.1016/j.exphem.2006.10.009 17258067 10.1016/j.exphem.2006.10.009 PMC1832107

[109] Vijay A, Inui T, Dodds M et al (2015) Factors that influence the extensional rheological property of saliva. PLoS One 10:e0135792. 10.1371/journal.pone.0135792 26305698 10.1371/journal.pone.0135792 PMC4549258

[110] Epstein JB, Thariat J, Bensadoun R-J et al (2012) Oral complications of cancer and cancer therapy: From cancer treatment to survivorship. CA Cancer J Clin 62:400–422. 10.3322/caac.21157 22972543 10.3322/caac.21157

[111] Tabak LA (1990) Structure And Function of Human Salivary Mucins. Crit Reviews Oral Biology Med 1:229–234. 10.1177/10454411900010040201 10.1177/10454411900010040201 2129627

[112] Faruque M, Wanschers M, Ligtenberg AJ et al (2022) A review on the role of salivary MUC5B in oral health. J Oral Biosci 64:392–399. 10.1016/j.job.2022.09.005 36206992 10.1016/j.job.2022.09.005

[113] Chaudhury NMA, Shirlaw P, Pramanik R et al (2015) Changes in Saliva Rheological Properties and Mucin Glycosylation in Dry Mouth. J Dent Res 94:1660–1667. 10.1177/0022034515609070 26446936 10.1177/0022034515609070

[114] Alliende C, Kwon Y-J, Brito M et al (2008) Reduced sulfation of MUC5B is linked to xerostomia in patients with Sjögren syndrome. Ann Rheum Dis 67:1480–1487. 10.1136/ard.2007.078246 17998215 10.1136/ard.2007.078246

[115] Aframian DJ, Keshet N, Nadler C et al (2019) Minor salivary glands: clinical, histological and immunohistochemical features of common and less common pathologies. Acta Histochem 121:151451. 10.1016/j.acthis.2019.151451 31653464 10.1016/j.acthis.2019.151451

[116] Kumar SS, Arany-Lao-Kan G, Xu X et al (2026) Minor salivary flow as a diagnostic tool for screening hyposalivation in medication-induced xerostomia. BMC Oral Health 26:454. 10.1186/s12903-026-07798-6 41639693 10.1186/s12903-026-07798-6 PMC12973732

[117] Wang Z, Li W, Hong X et al (2016) Minor salivary glands function is decreased in hyposalivation-related diseases. Arch Oral Biol 69:63–70. 10.1016/j.archoralbio.2016.05.012 27243418 10.1016/j.archoralbio.2016.05.012

[118] Carvalho IH, Barros EF, de Lima BH et al (2025) Photobiomodulation as a therapeutic alternative to improve xerostomia and hyposalivation in a Sjögren’s disease patient: case report. Lasers Dent Sci 9:5. 10.1007/s41547-025-00279-z

[119] Sanchez-Ramirez C, Bernotti AL, De Jesús E et al (2026) Diode-laser-assisted biopsy and photobiomodulation therapy in the management of Sjögren’s disease: a case report. J Med Case Rep 20:228. 10.1186/s13256-026-05931-1 41866508 10.1186/s13256-026-05931-1 PMC13130530

[120] Lopez-Garzon M, Plata-Peregrina M, del Perez-Sanchez C EI, et al (2025) Photobiomodulation for restoring salivary flow after radiotherapy in head and neck cancer: a randomised placebo-controlled trial. BMC Oral Health 25:1468. 10.1186/s12903-025-06735-3 41013422 10.1186/s12903-025-06735-3 PMC12465775

[121] Arbabi-Kalati F, Arbabi-Kalati F, Moridi T (2013) Evaluation of the effect of low level laser on prevention of chemotherapy-induced mucositis. Acta Med Iran 51:157–162 23605599

[122] Caillot E, Denis F (2012) Radio-induced oral and pharyngeal mucositis: management updates. Cancer Radiother 16:358–363. 10.1016/j.canrad.2012.05.005 22841560 10.1016/j.canrad.2012.05.005

[123] Durães CP, Fonseca LL, Santos SHS et al (2025) Effect of photobiomodulation on rat parotid gland cells after ionizing radiation – an experimental study in animal model – Part 1. J Stomatol Oral Maxillofac Surg 126:102485. 10.1016/j.jormas.2025.102485 40615114 10.1016/j.jormas.2025.102485

[124] Louzeiro GC, Teixeira Dda, Cherubini S K, et al (2020) Does laser photobiomodulation prevent hyposalivation in patients undergoing head and neck radiotherapy? A systematic review and meta-analysis of controlled trials. Crit Rev Oncol Hematol 156:103115. 10.1016/j.critrevonc.2020.103115 33039721 10.1016/j.critrevonc.2020.103115

[125] Schepanski N, da Silva Liebl BM, Mendes RT et al (2025) Effects of photobiomodulation and bethanechol chloride treatment on salivary composition and flow rate in head and neck cancer patients undergoing radiotherapy: longitudinal interventional experimental clinical study. Support Care Cancer 33:113. 10.1007/s00520-024-09120-y 39825016 10.1007/s00520-024-09120-y

[126] El Mobadder M, Farhat F, El Mobadder W, Nammour S (2018) Photobiomodulation therapy in the treatment of oral mucositis, dysgeusia and oral dryness as side-effects of head and neck radiotherapy in a cancer patient: a case report. Dent J (Basel) 6:64. 10.3390/dj6040064 30423851 10.3390/dj6040064 PMC6313426

[127] Mosannen Mozaffari P, Delavarian Z, Fekrazad R et al (2024) Evaluation of the effect of photobiomodulation on radiation-induced xerostomia in head and neck cancer patients: a randomized clinical trial. J Lasers Med Sci 15:e4. 10.34172/jlms.2024.04 38655042 10.34172/jlms.2024.04 PMC11033859

[128] Schepanski N, Costa FS, Machado EFM et al (2024) Evaluation of photobiomodulation therapy (PBMT) on salivary flow and composition in head and neck cancer patients undergoing radiation therapy. Oral Surg Oral Med Oral Pathol Oral Radiol 137:253–263. 10.1016/j.oooo.2023.11.007 38218654 10.1016/j.oooo.2023.11.007

[129] Palma LF, Gonnelli FAS, Marcucci M et al (2017) Impact of low-level laser therapy on hyposalivation, salivary pH, and quality of life in head and neck cancer patients post-radiotherapy. Lasers Med Sci 32:827–832. 10.1007/s10103-017-2180-3 28258315 10.1007/s10103-017-2180-3

[130] Alsoghier A, Mutaieb S, Bukhari A et al (2025) Management of radiotherapy-induced hyposalivation using photobiomodulation therapy: a case series. Lasers Med Sci 40:25. 10.1007/s10103-025-04292-3 39833441 10.1007/s10103-025-04292-3

[131] de Carvalho ESRM, Mendes FM, Degasperi GR, Pinheiro SL (2023) Photobiomodulation for the management of xerostomia and oral mucositis in patients with cancer: a randomized clinical trial. Lasers Med Sci 38:101. 10.1007/s10103-023-03760-y 37060370 10.1007/s10103-023-03760-y

[132] Borghesi Brunelli G, Pinheiro SL (2025) Combined extraoral and intraoral photobiomodulation for the prevention and management of oral mucositis and Xerostomia caused by the treatment of head and neck cancers. Lasers Med Sci 40:295. 10.1007/s10103-025-04539-z 40549226 10.1007/s10103-025-04539-z

[133] Melo WWP, Aragão WAB, Ferreira MKM et al (2026) Effects of Clarified Açaí Supplementation and Photobiomodulation on the Parotid Glands of Rats Submitted to Chemotherapy. J Oral Pathol Med 55:120–132. 10.1111/jop.70072 41133718 10.1111/jop.70072 PMC12774578

[134] Khalil M, Hamadah O, Saifo M (2025) Photobiomodulation preconditioning for oral mucositis prevention and quality of life improvement in chemotherapy patients: a randomized clinical trial. BMC Oral Health 25:190. 10.1186/s12903-025-05579-1 39910516 10.1186/s12903-025-05579-1 PMC11800611

[135] Simões A, Platero MD, Campos L et al (2009) Laser as a therapy for dry mouth symptoms in a patient with Sjögren’s syndrome: a case report. Spec Care Dentist 29:134–137. 10.1111/j.1754-4505.2009.00078.x 19938253 10.1111/j.1754-4505.2009.00078.x

[136] Cafaro A, Arduino PG, Gambino A et al (2015) Effect of laser acupuncture on salivary flow rate in patients with Sjögren’s syndrome. Lasers Med Sci 30:1805–1809. 10.1007/s10103-014-1590-8 24820476 10.1007/s10103-014-1590-8

[137] Fidelix T, Czapkowski A, Azjen S et al (2018) Low-level laser therapy for xerostomia in primary Sjögren’s syndrome: a randomized trial. Clin Rheumatol 37:729–736. 10.1007/s10067-017-3898-9 29119483 10.1007/s10067-017-3898-9

[138] Colares DF, de Souto Medeiros MR, de Araújo ACG et al (2025) Photobiomodulation therapy for salivary gland hypofunction: a preliminary human study. Lasers Med Sci 40:264. 10.1007/s10103-025-04512-w 40488944 10.1007/s10103-025-04512-w

[139] Mercadante V, Jensen SB, Smith DK et al (2021) Salivary Gland Hypofunction and/or Xerostomia Induced by Nonsurgical Cancer Therapies: ISOO/MASCC/ASCO Guideline. J Clin Oncol 39:2825–2843. 10.1200/JCO.21.01208 34283635 10.1200/JCO.21.01208

[140] Wolff A, Fox C, Porter P, Konttinen ST Y (2012) Established and Novel Approaches for the Management of Hyposalivation and Xerostomia. Curr Pharm Des 18:5515–5521. 10.2174/138161212803307509 22632391 10.2174/138161212803307509

[141] Carrara-Cotomacio C, Campos L, Simoes A et al (2016) Influence of bethanechol on salivary parameters in irradiated patients. Med Oral Patol Oral Cir Bucal. 10.4317/medoral.21395 10.4317/medoral.21395 PMC5217501 27918737

[142] Jham BC, Teixeira IV, Aboud CG et al (2007) A randomized phase III prospective trial of bethanechol to prevent radiotherapy-induced salivary gland damage in patients with head and neck cancer. Oral Oncol 43:137–142. 10.1016/j.oraloncology.2006.01.013 16798061 10.1016/j.oraloncology.2006.01.013

[143] Brizel DM, Wasserman TH, Henke M et al (2000) Phase III Randomized Trial of Amifostine as a Radioprotector in Head and Neck Cancer. J Clin Oncol 18:3339–3345. 10.1200/JCO.2000.18.19.3339 11013273 10.1200/JCO.2000.18.19.3339

[144] Vivino FB, Carsons SE, Foulks G et al (2016) New Treatment Guidelines for Sjögren’s Disease. Rheumatic Disease Clin North Am 42:531–551 10.1016/j.rdc.2016.03.010 PMC5812283 27431353

[145] Tsai R-Y, Lin S-Y, Chen C-C, Hsiao Y (2025) Efficacy of Acupuncture in Managing Radiation-Induced Xerostomia: An Updated Meta-Analysis. Int J Med Sci 22:2802–2815. 10.7150/ijms.110366 40520905 10.7150/ijms.110366 PMC12163620

[146] Martins ED, Rebouças NIC, Dos Santos SQM et al (2026) Efficacy of artificial saliva in preventing oral changes in patients admitted to the ICU: a controlled clinical trial. Clin Oral Investig. 10.1007/s00784-026-06864-1 42026217 10.1007/s00784-026-06864-1 PMC13106235

[147] Topol EJ (2019) High-performance medicine: the convergence of human and artificial intelligence. Nat Med 25:44–56. 10.1038/s41591-018-0300-7 30617339 10.1038/s41591-018-0300-7

[148] Pringle S, Van Os R, Coppes RP (2013) Concise review: adult salivary gland stem cells and a potential therapy for xerostomia. Stem Cells 31:613–619. 10.1002/stem.1327 23335219 10.1002/stem.1327

[149] Park H-J, Cho J, Oh S-J, Kim H-S (2025) Organoid-based modeling and regenerative strategies for salivary gland dysfunction. Korean J Physiol Pharmacol 29:683–698. 10.4196/kjpp.25.149 40776800 10.4196/kjpp.25.149 PMC12576417

[150] Klangprapan J, Souza GR, Ferreira JN (2024) Bioprinting salivary gland models and their regenerative applications. BDJ Open 10:39. 10.1038/s41405-024-00219-2 38816372 10.1038/s41405-024-00219-2 PMC11139920

[151] Ozdemir T, Fowler EW, Hao Y et al (2016) Biomaterials-based strategies for salivary gland tissue regeneration. Biomater Sci 4:592–604. 10.1039/C5BM00358J 26878077 10.1039/c5bm00358j PMC4803517

[152] Shrateh ON, Habib A, Ur Rehman Shamsi H et al (2026) Treatment options for radiation-induced xerostomia in patients with head and neck cancer: a systematic review and meta-analysis. Br J Oral Maxillofac Surg 64:24–32. 10.1016/j.bjoms.2025.10.003 41253620 10.1016/j.bjoms.2025.10.003

[153] Bilal MU, Alvi S, Humayune F et al (2026) Advances in gene therapy for xerostomia: current perspectives and future directions: a narrative review. Health Sci Rep. 10.1002/hsr2.72295 42079712 10.1002/hsr2.72295 PMC13129664

